# Supplementary material for: Human cortical networks trade communication efficiency for computational reliability
Source: Sci Adv. 2026 Sep 2;12(36):eaef2894. doi: 10.1126/sciadv.aef2894 (PMC13537246; doi:10.1126/sciadv.aef2894)
Supplement: Supplementary file 1 [file sciadv.aef2894_sm.pdf]

Science Advances
AAAS

Supplementary Materials for

Human cortical networks trade communication efficiency for computational reliability

Kayson Fakhar *et al.*

Corresponding author: Kayson Fakhar, kayson.fakhar@mrc-cbu.cam.ac.uk

Sci. Adv. **12**, eaef2894 (2026)
DOI: 10.1126/sciadv.aef2894

This PDF file includes:

Figs. S1 to S12
Table S1

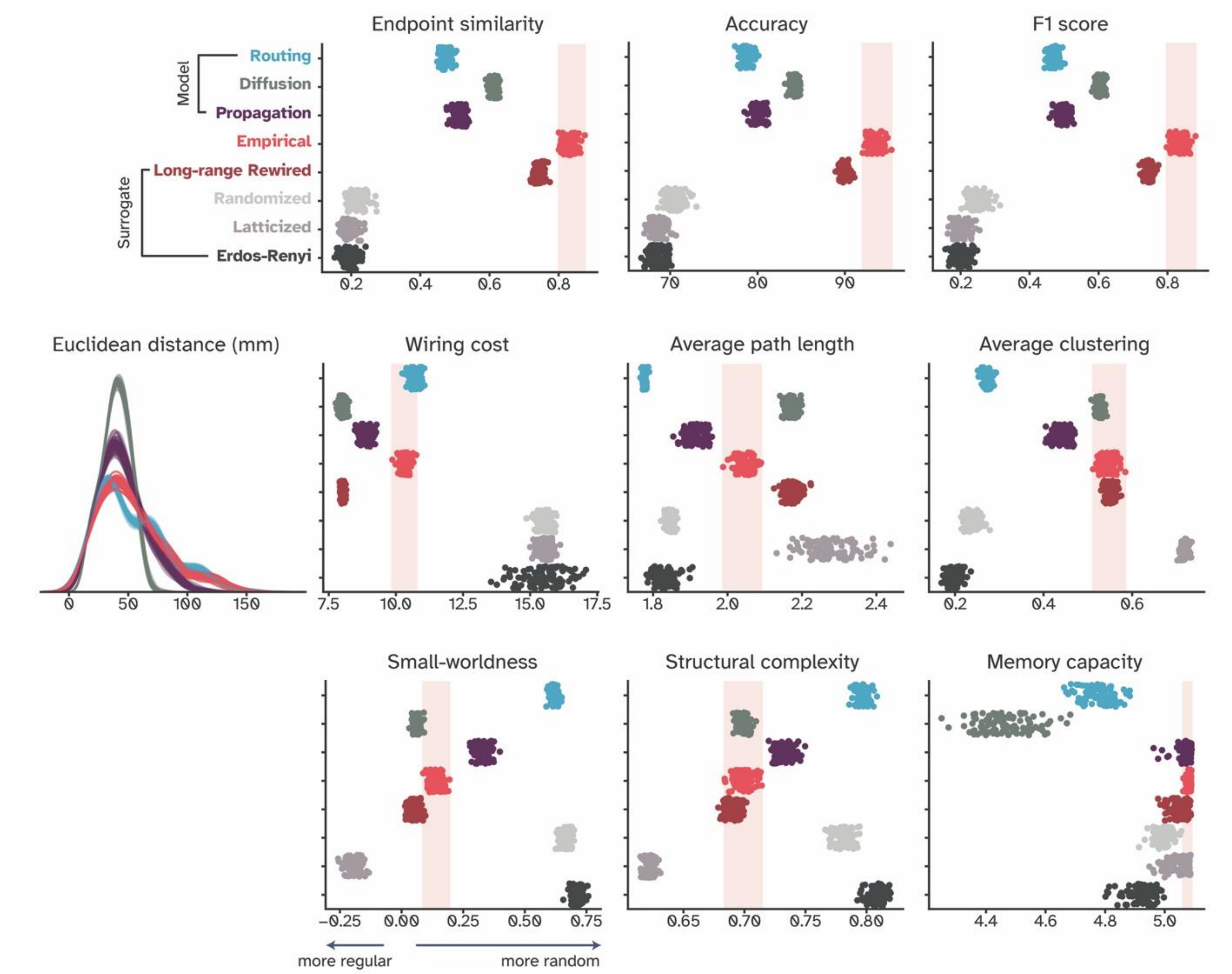

Fig. S1. Replicating the main findings using Desikan-Killiany atlas of cortical parcellation.

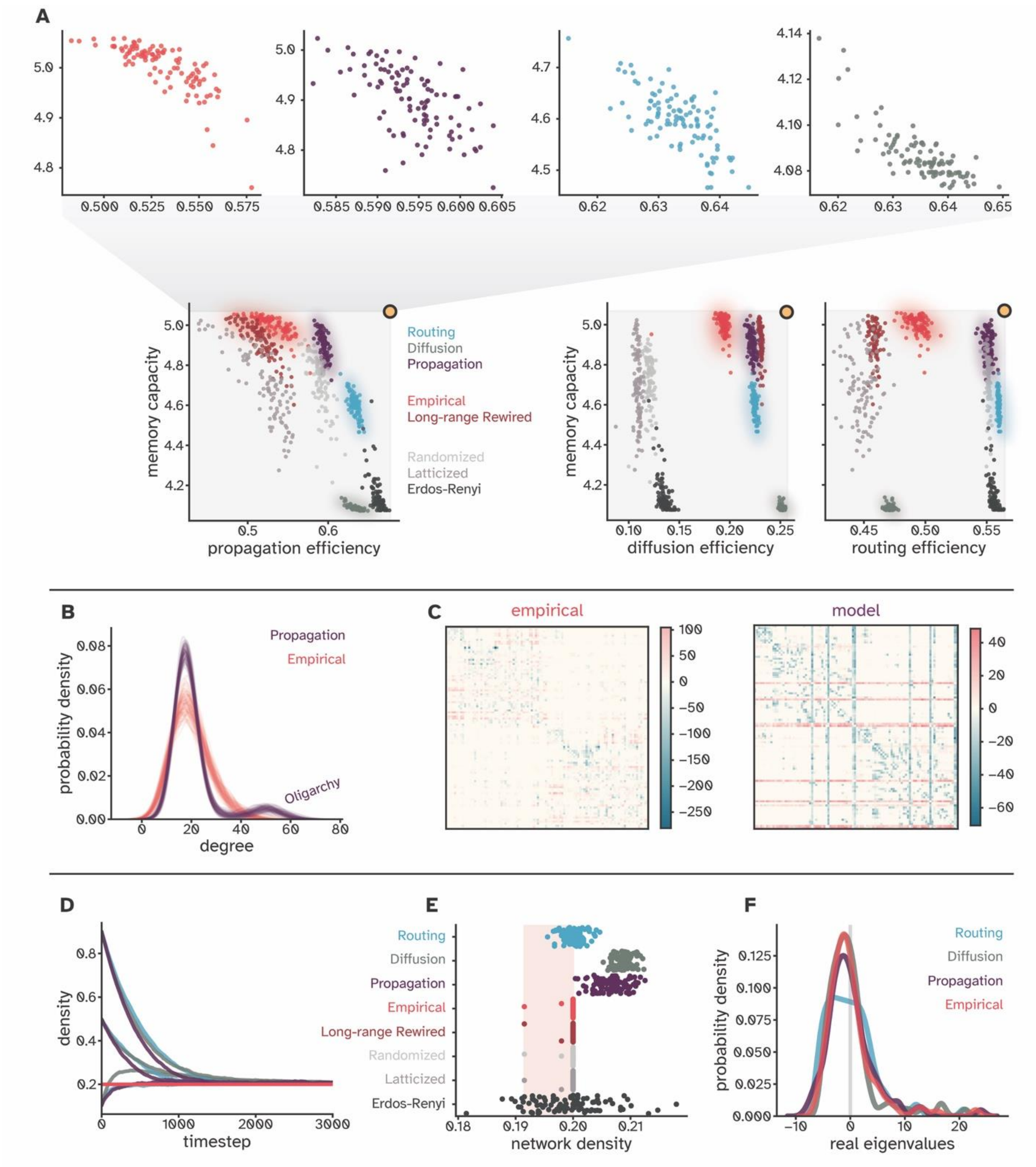

Fig. S2. Extended analyses of model networks.

(A) Relationship between memory capacity and efficiency measures under different communication regimes. **(B)** Degree distributions show the emergence of a hyper-connected oligarchy in propagation-based models compared to the empirical cortex. **(C)** Cost-benefit maps show how model networks asymmetrically concentrate resources into oligarchic hubs, unlike the more distributed empirical pattern. **(D)** Developmental trajectories of network density across different random initialization reveal convergence toward a stable equilibrium. **(E)** Final network

densities closely match empirical targets with ±0.01 error margin. **(F)** Spread of real eigenvalues that is measured in structural complexity for empirical and model networks.

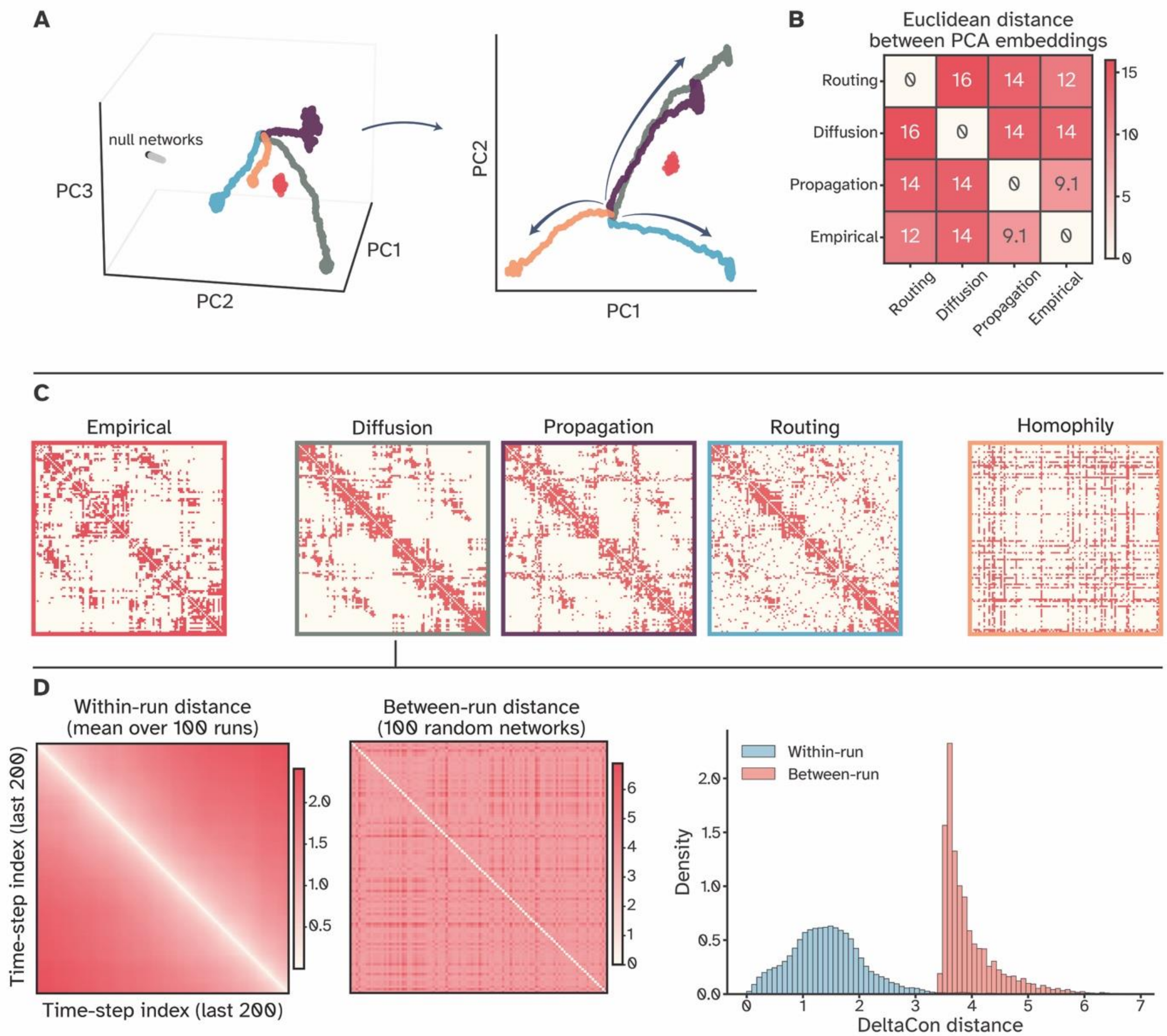

Fig. S3. PCA-based embeddings of networks.

(A) Joint PCA embedding of networks shows the developmental trajectories they take and their settle points. **(B)** Pairwise Euclidean distances between these points quantify the relative separation of network types, showing closer alignment of propagation-based networks with empirical data. **(C)** Connectivity matrices illustrate wiring patterns of empirical, diffusion-, propagation-, and routing-based networks, compared with a homophily-driven model. Homophily produces an extreme core–periphery structure, distinct from both empirical and communication-based networks. **(D)** Robustness of the converged configurations to initial conditions. The model was run from 100 distinct random-graph initializations spanning densities of 0.1–0.9 and refitted to the empirical connectome in each case. For every run, the DeltaCon distance between converged networks (between-run) was compared to the DeltaCon distance accumulated by each run across its final 200 optimization steps (within-run). Between-run distances were approximately threefold larger than within-run drift, and the two distributions were non-overlapping, indicating that the model settles into a field of stable configurations rather than a single attractor. In other words, across different instances, macroscopic statistics are highly similar while microstructural details vary.

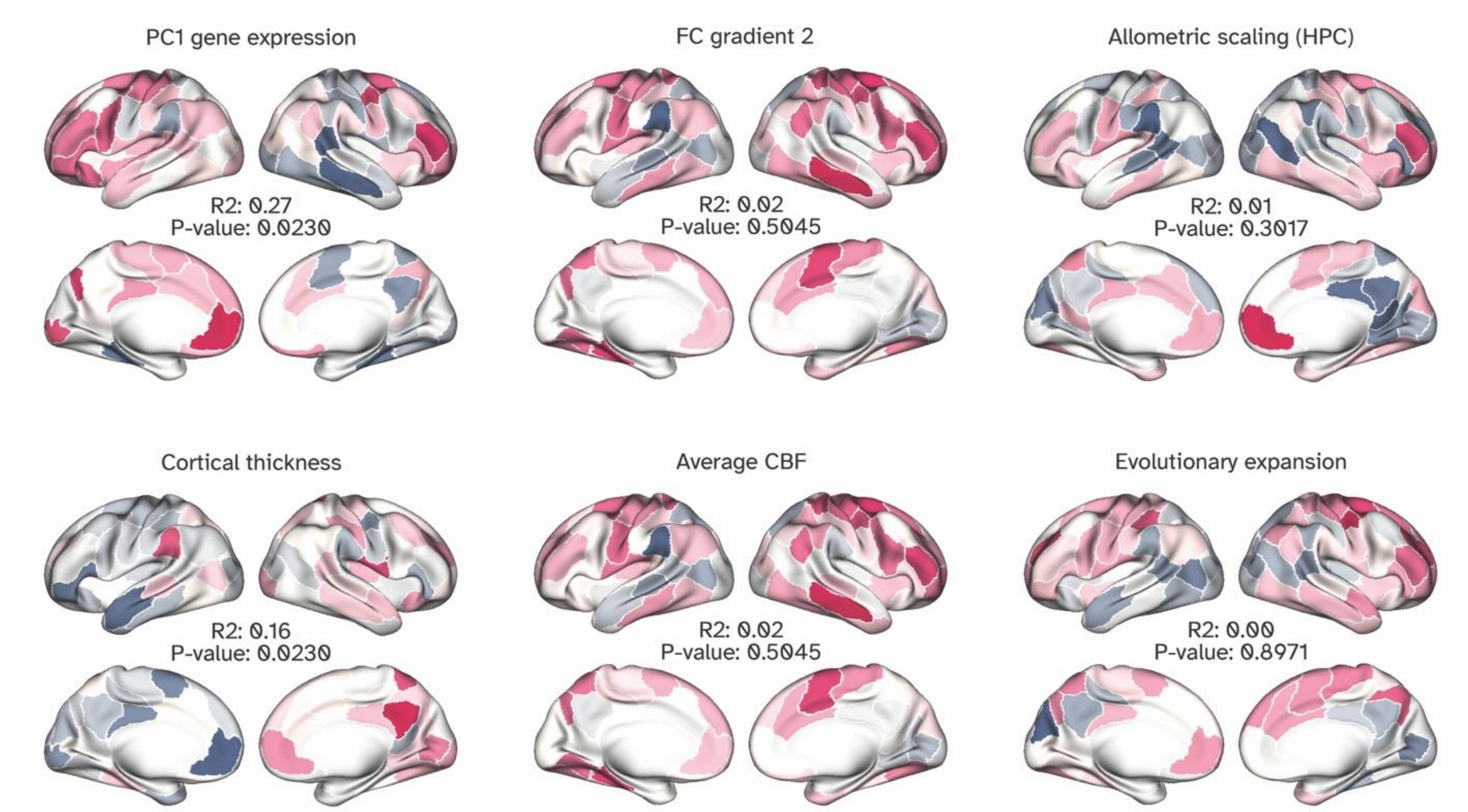

Fig S4. Comparison of payoff dynamics with other cortical maps. These maps are explained in detail elsewhere (*55*). However, briefly, the gene expression map is constructed from the first principal component of the Allen Institute's Human Brain atlas (*102*). The second gradient of FC is from (*52*), the allometric scaling represents the scaling of individual regions in a healthy population (*103*), map of cortical thickness is from (*104*), average CBF represents the regional cerebral blood perfusion and is from (*102*). Lastly, map of evolutionary expansion is from (*55*).

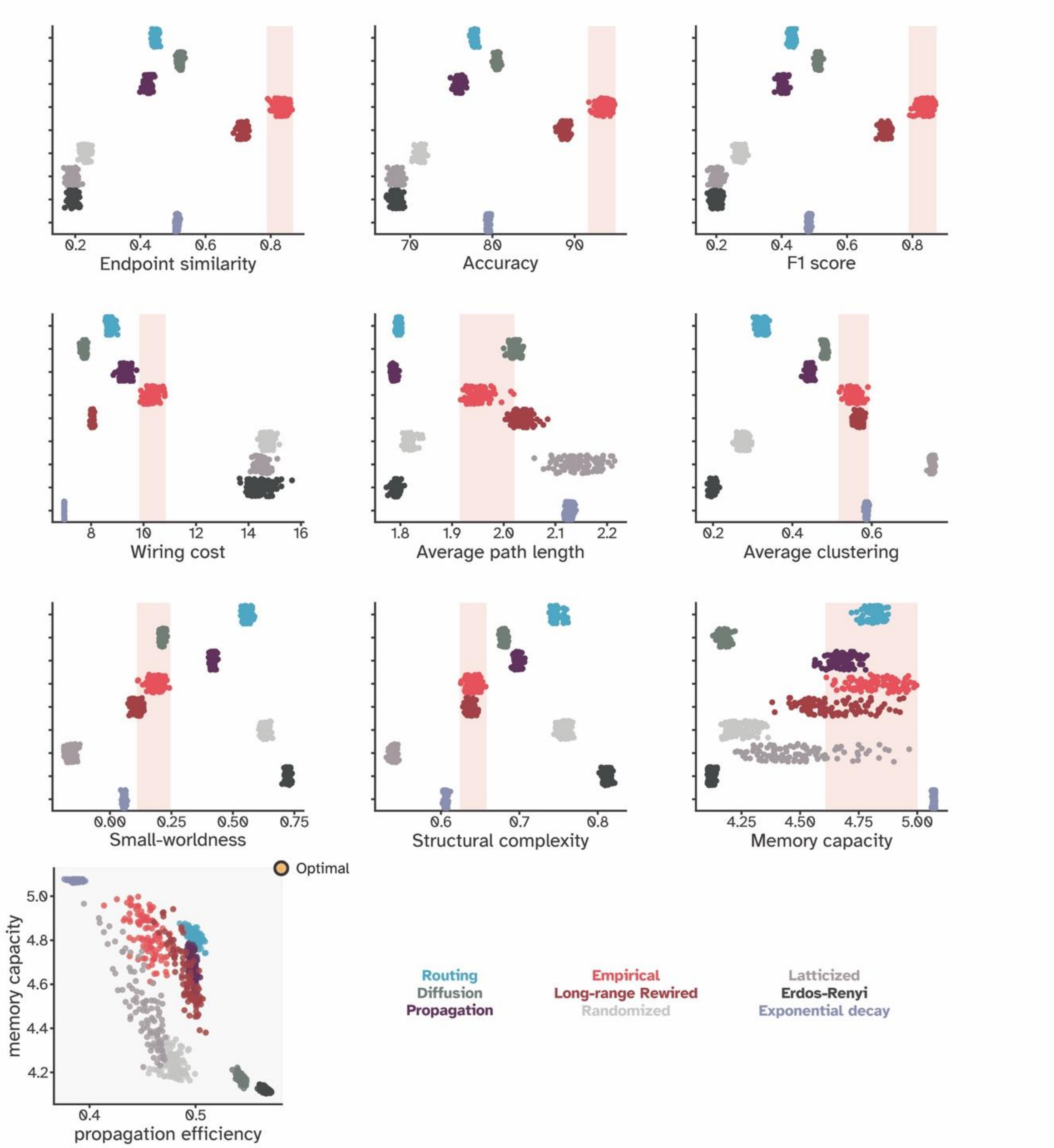

Fig. S5. Replication of the main findings with an independent dataset and an additional null model, i.e., based on the exponential decay rule.

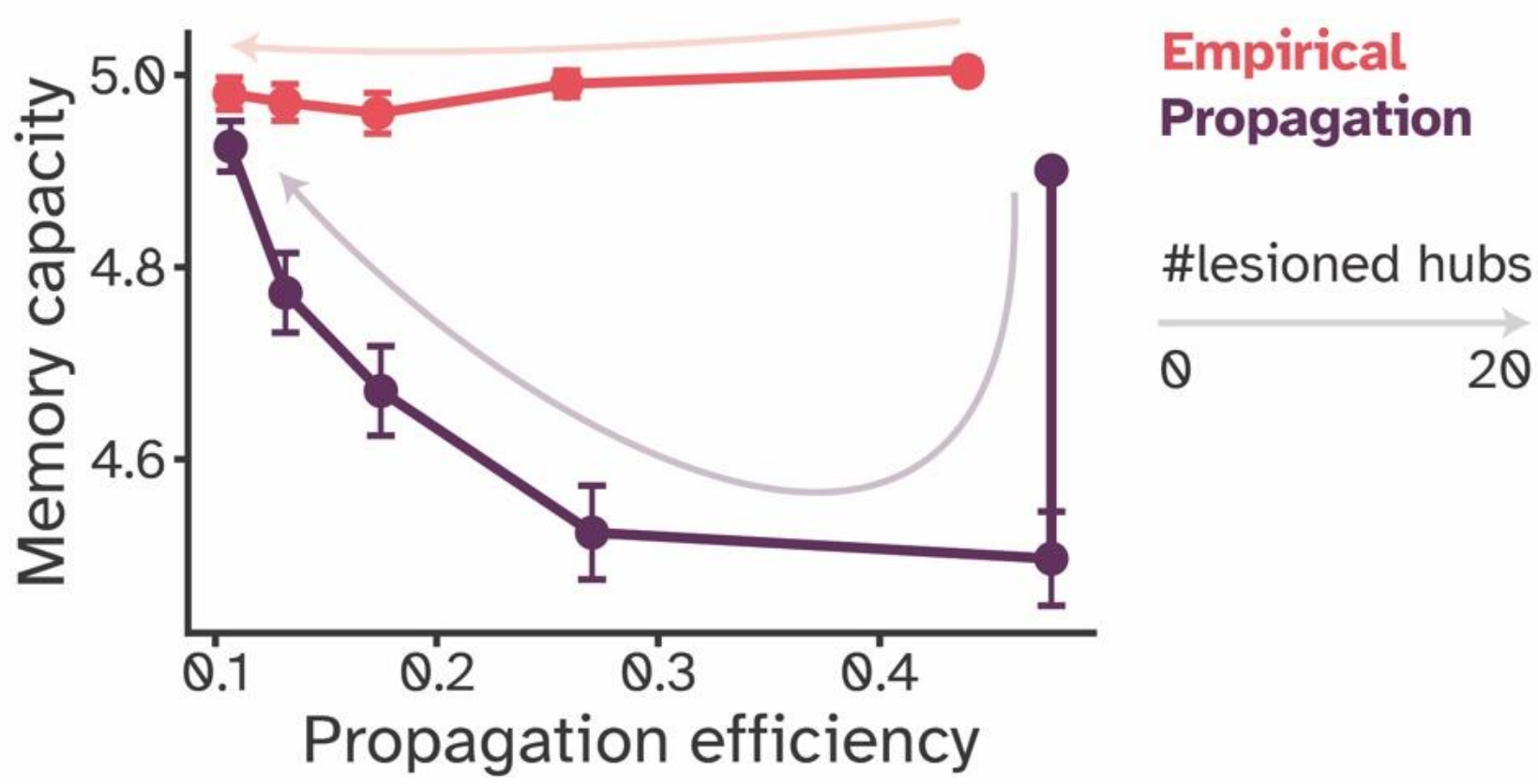

Fig. S6. Contextualizing the effect of node removal with respect to its effect on the memory capacity-propagation efficiency plane.

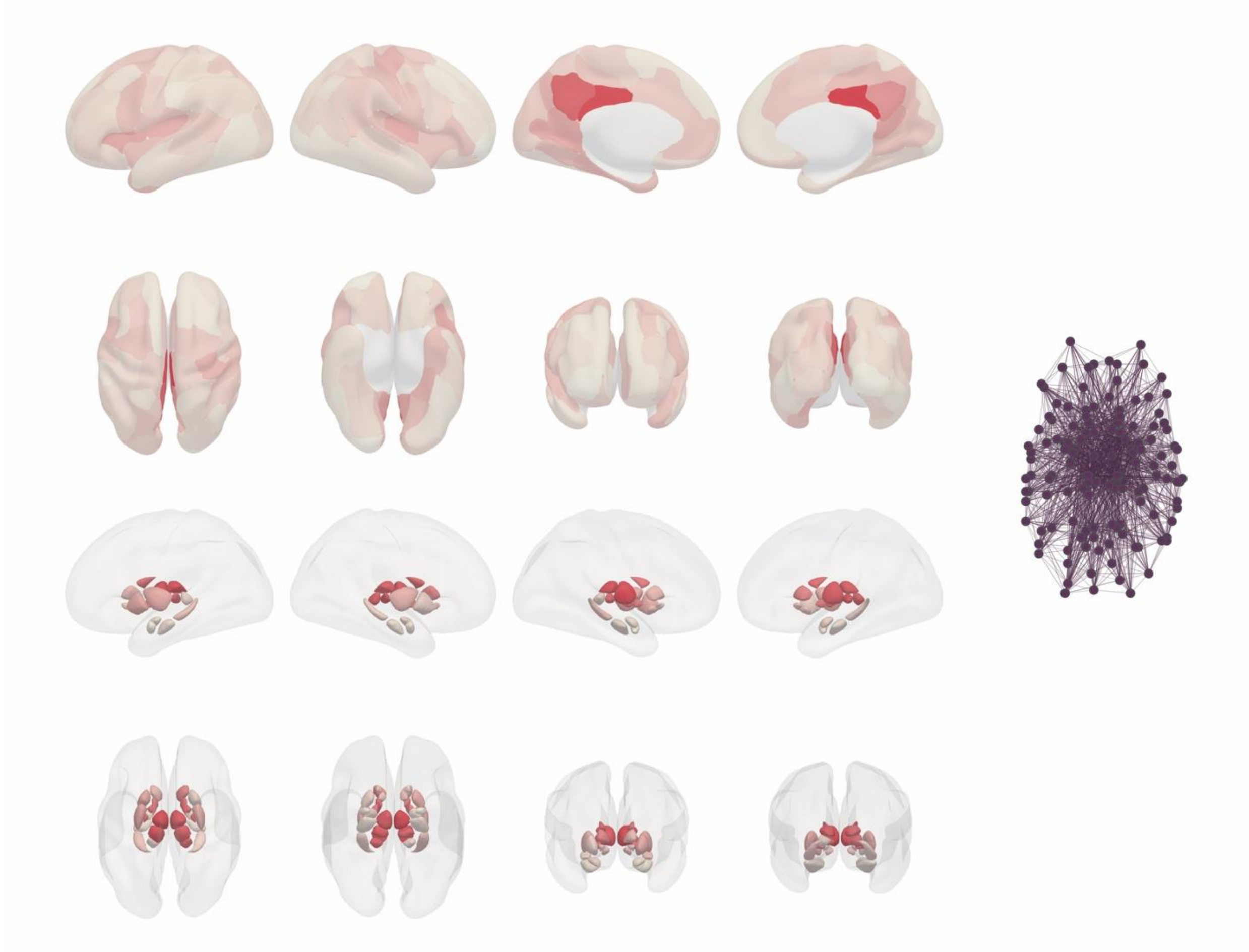

Fig. S7. Subcortical extension of the oligarchic cortical organization.

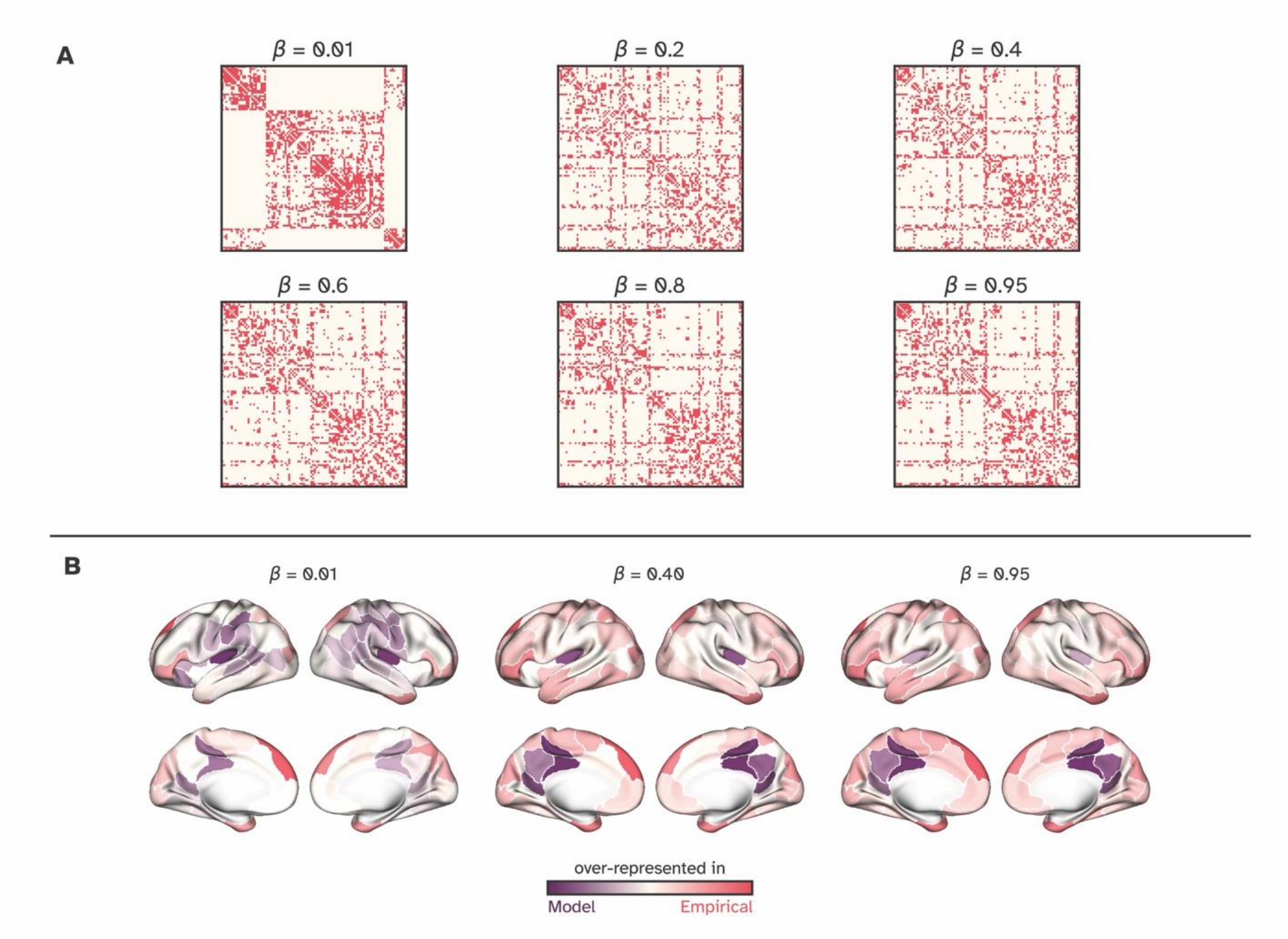

Fig. S8. Influence of propagation strength on the spatial and network organization of the oligarchic regions.

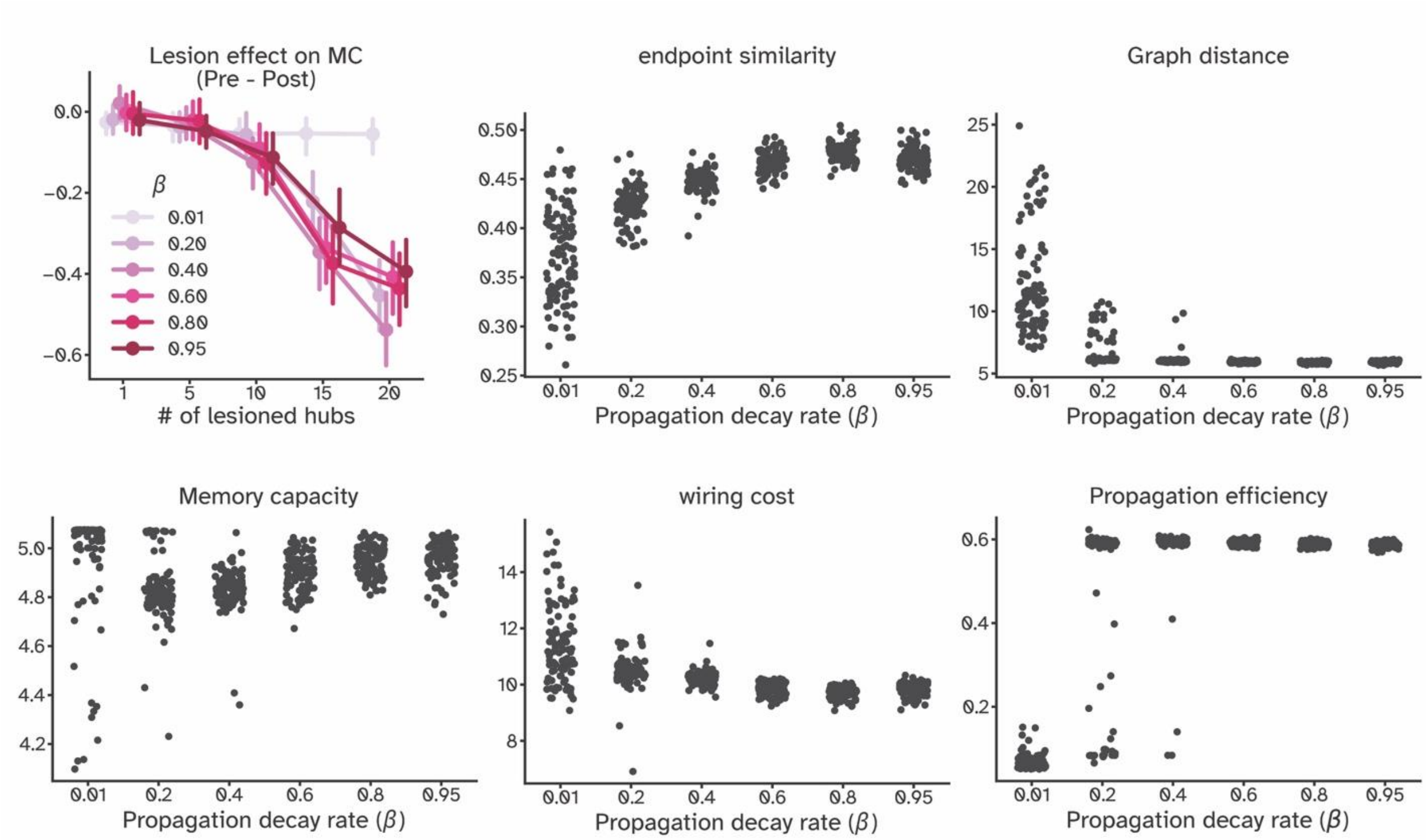

Fig. S9. Influence of propagation strength on different network measures. Companion to Fig. S8, which examined the spatial and network organization of oligarchic regions as a function of propagation strength. Here, the propagation strength was varied across its admissible range, networks were re-generated under the propagation regime and different measures were recomputed. Across a wide range of 0.2 – 0.95, these measures show little variability, further supporting that the findings related to oligarchic organization are not an artifact of propagation strength.

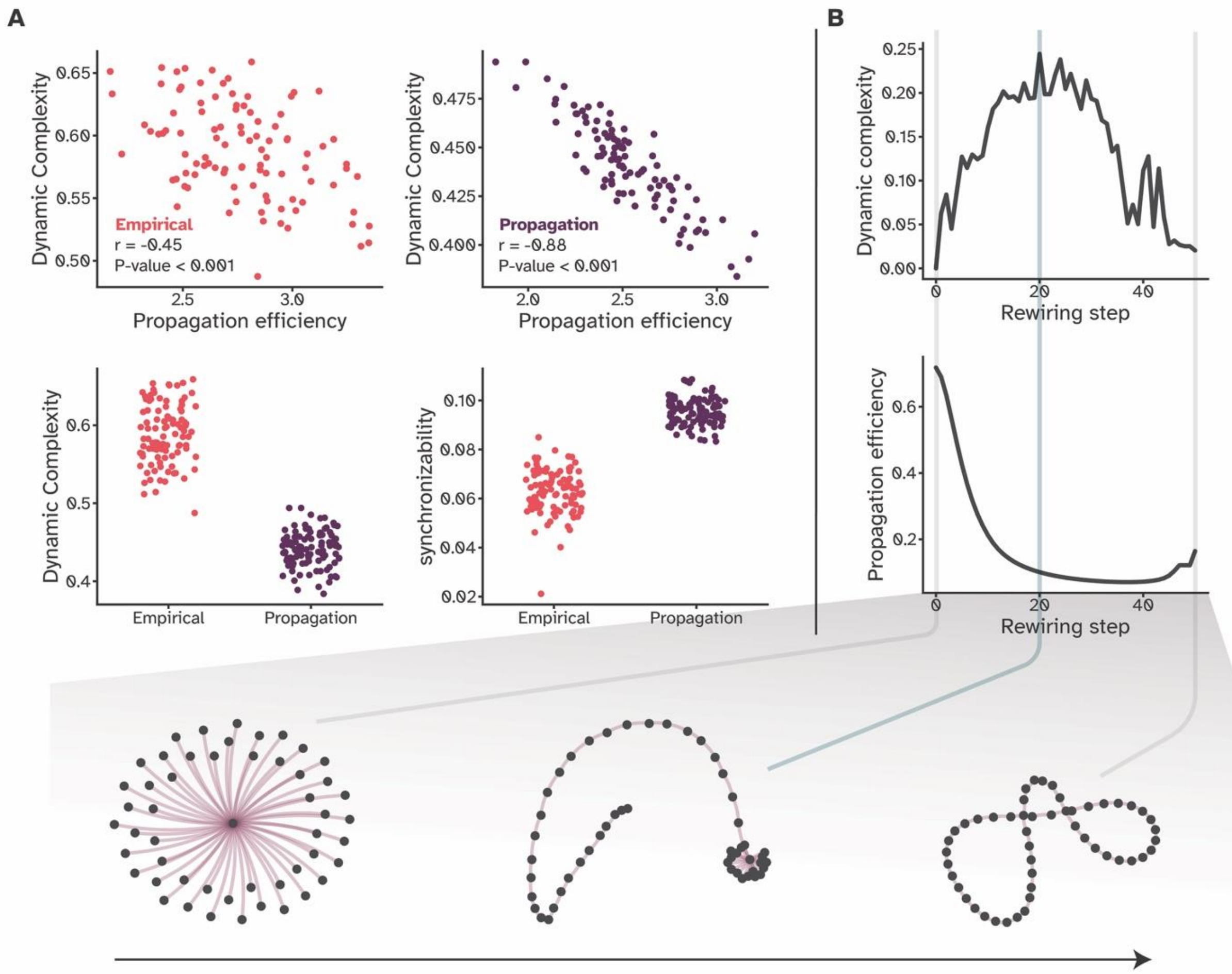

Fig. S10. Replicating the computation-communication trade-off using dynamic complexity as a proxy for cortical computation. (A) Dynamic complexity is inversely correlated with propagation efficiency across networks, and this correlation is roughly twice as strong in optimal propagation networks as in the empirical human brain. Dynamic complexity is higher in empirical networks, while readiness for synchronizability is higher in propagation-based optimal networks. We hypothesized that networks with greater communication efficiency, by definition, synchronize more easily, and that beyond a certain point this homogenizes signal propagation and limits the encoding of complex representations. **(B)** A toy example illustrates this trade-off: rewiring a hyper-efficient graph (one hub, all others peripheral) into a grid (no hubs) shows that propagation efficiency peaks at the former configuration, where dynamic complexity is lowest, while dynamic complexity peaks at hybrid configurations with a looser core–periphery relationship.

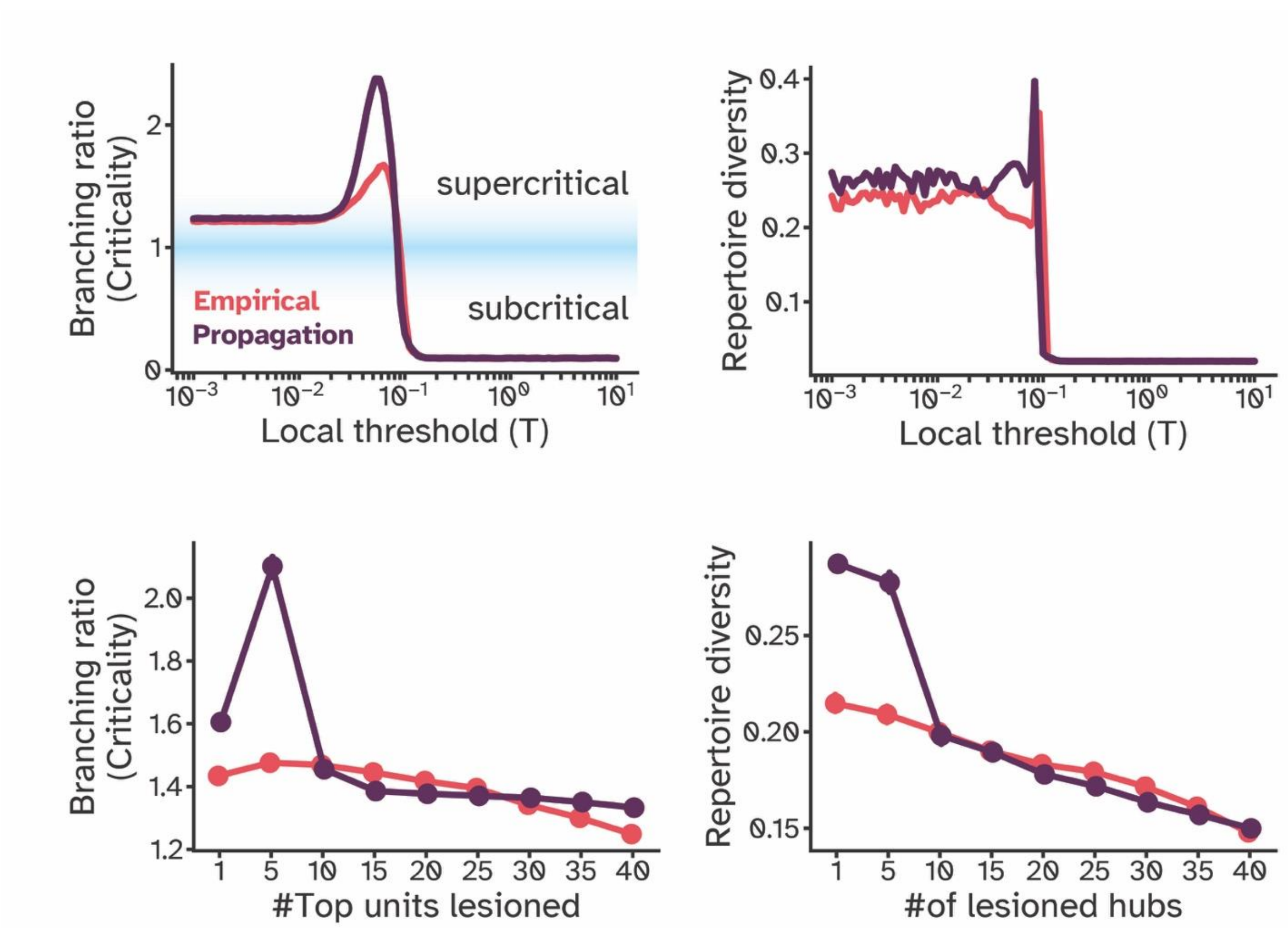

Fig. S11. Replicating the fragility of optimal communication networks against targeted attacks using criticality and repertoire diversity as proxies for cortical computation. Parallel to the progressive-lesioning analysis in Fig. 3C, where memory capacity served as the readout of cortical computation. Here, the same targeted-attack was applied to empirical and propagation-based networks, but task performance was replaced by two dynamics-based proxies: (i) a criticality index quantifying proximity to the edge between ordered and chaotic regimes, and (ii) the diversity of the network's dynamical repertoire under stochastic input. Empirical networks degrade gracefully on both measures, whereas propagation-based networks show a sharp collapse once the small oligarchic core is perturbed, reproducing the asymmetry observed with memory capacity.

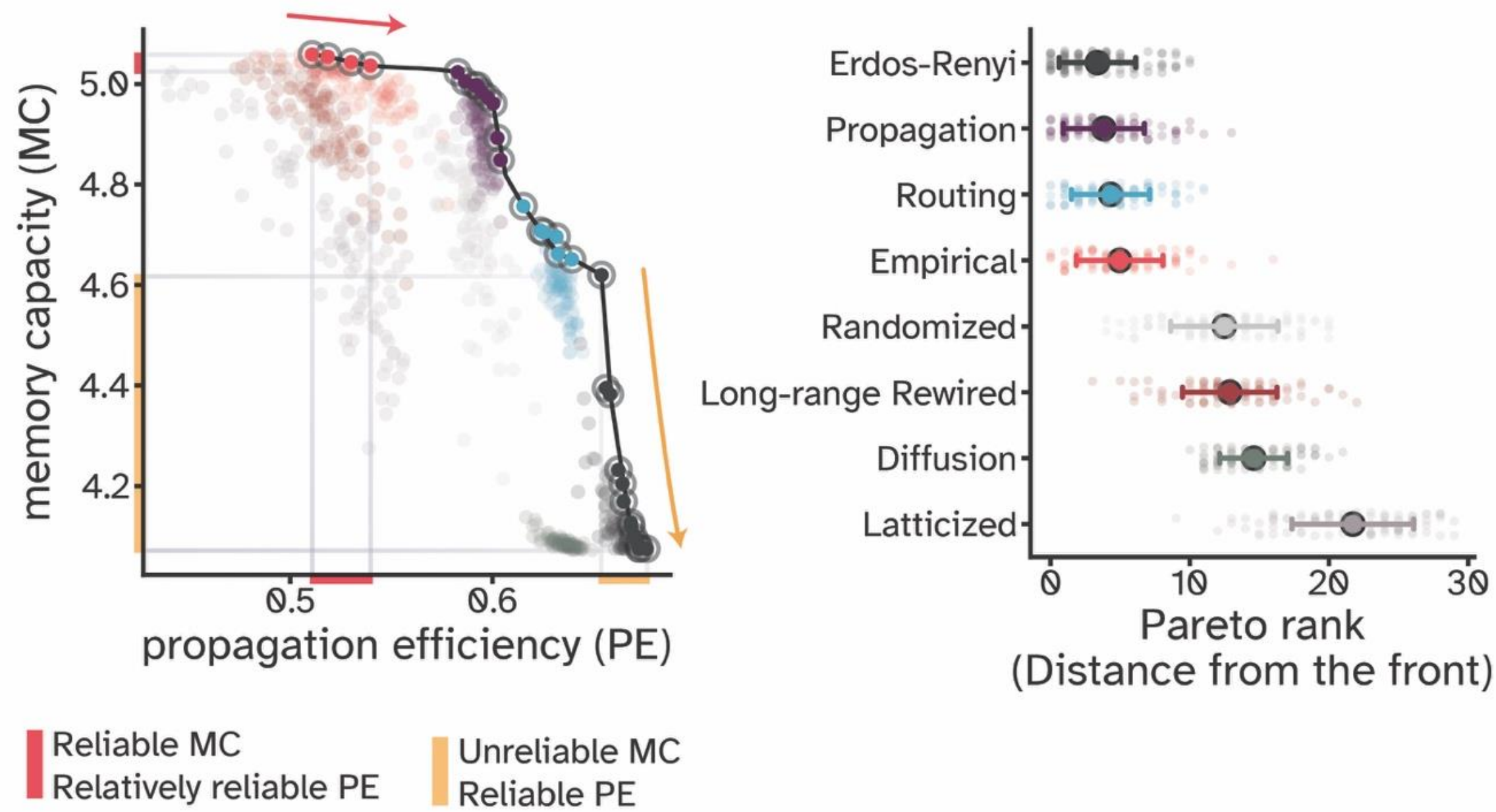

Fig. S12. A more principled approach towards quantifying the observed pareto-optimality. Formalization of the qualitative Pareto-front argument in Fig. 3A, where memory capacity and global propagation efficiency were plotted against each other and the empirical brain was shown to occupy a non-dominated region of the solution space. Here, the Pareto front was identified across the full population of empirical, generative, and null networks as following: a network is retained on the front if no other network simultaneously achieves higher memory capacity and higher propagation efficiency. Distance to the front was then computed for every network, providing a continuous, network-agnostic measure of Pareto-optimality. Empirical cortical networks lie on or within a narrow band of the front where different instances show moderate variability in propagation efficiency but minimal for memory capacity, making them functionally reliable. In contrast, Erdos-Renyi (fully random) networks show highly volatile memory capacity performance while keeping a stable propagation efficiency, which makes them functionally unreliable.

action	eating	insight	naming	semantic memory
adaptation	efficiency	integration	navigation	sentence comprehension
addiction	effort	intelligence	object recognition	skill
anticipation	emotion	intention	pain	sleep
anxiety	emotion regulation	interference	perception	social cognition
arousal	empathy	judgment	planning	spatial attention
association	encoding	knowledge	priming	speech perception
attention	episodic memory	language	psychosis	speech production
autobiographical memory	expectancy	language comprehension	reading	strategy
balance	expertise	learning	reasoning	strength
belief	extinction	listening	recall	stress
categorization	face recognition	localization	recognition	sustained attention
cognitive control	facial expression	loss	rehearsal	task difficulty
communication	familiarity	maintenance	reinforcement learning	thought
competition	fear	manipulation	response inhibition	uncertainty
concept	fixation	meaning	response selection	updating
consciousness	focus	memory	retention	utility
consolidation	gaze	Memory retrieval	retrieval	valence
context	goal	mental imagery	reward anticipation	verbal fluency
coordination	hyperactivity	monitoring	rhythm	visual attention
decision	imagery	mood	risk	visual perception
decision making	impulsivity	morphology	rule	word recognition
detection	induction	motor control	salience	working memory
discrimination	inference	movement	search	distraction
inhibition	multisensory	selective attention		

Supplementary Table 1: List of 123 cognitive tasks that are used in the Neurosynth PC1 map (*100*).
